# Transcriptomic and Metabolomic Joint Analysis Revealing Different Metabolic Pathways and Genes Dynamically Regulating Bitter Gourd (*Momordica charantia* L.) Fruit Growth and Development in Different Stages

**DOI:** 10.3390/plants14142248

**Published:** 2025-07-21

**Authors:** Boyin Qiu, Dazhong Li, Qianrong Zhang, Hui Lin, Yongping Li, Qingfang Wen, Haisheng Zhu

**Affiliations:** Crops Research Institute, Fujian Academy of Agricultural Sciences, Fuzhou 350002, China; qby747_1030@163.com (B.Q.); fjfzldz@163.com (D.L.); zhqrong@163.com (Q.Z.); lhlzl540@163.com (H.L.); lyp_lily@163.com (Y.L.)

**Keywords:** bitter gourd (*Momordica charantia* L.), fruit, growth and development, omics, gene expression

## Abstract

Insights into dynamic regulatory factors in various stages of growth and development can guide strategies for precision and targeted breeding. Bitter gourd, as a vegetable product with medicinal value, plays a role in both agricultural and medical fields. In this study, phenotypic observations, metabolomic and transcriptomic analyses, and differential gene expression patterns, along with a correlation analysis, were conducted in different stages of fruit growth and development. The results revealed that the growth rate of fruit’s fresh weight, length, diameter, and flesh thickness during the first seven days was slow, and that it then rapidly increased after the seventh day, and finally slowed once more after 17 days, indicating that the overall process followed a “slow–fast–slow” pattern. Transcriptomic and metabolomic analyses identified several differentially expressed genes and metabolites, and joint analyses revealed that each of the glycolysis/gluconeogenesis, fructose and mannose metabolism and flavonoid biosynthesis pathways individually play significant roles in the dynamic regulation of fruit growth and development during the early, middle, and late stages. Among these, 53 differentially expressed genes (DEGs) and 12 differentially expressed metabolites (DEMs) were found in these pathways. A total of 12 randomly selected DEGs were analyzed using quantitative PCR, and the results showed that gene expression levels were generally consistent with transcriptomic sequencing results, exhibiting dynamic changes with varying expression levels. Correlation analysis revealed that 11 DEMs were positively correlated with four traits except for arbutin, while eight DEGs were related to all traits, including six significantly positive and two significantly negative correlations. These findings enhance our understanding of the regulatory network governing yield and quality and provide substantial evidence to support improvements in breeding programs.

## 1. Introduction

Bitter gourd (*Momordica charantia* L.), a plant from the genus Momordica within the Cucurbitaceae family, is an annual climbing and weakly herbaceous species. Native to East India, it is widely cultivated in tropical to temperate regions globally, including common cultivation in both northern and southern China, for its wide fruiting period spanning from May to October [1,2]. Known for its bitter taste, its fruit is not only a vegetable product but also one with medicinal value, being rich in proteins, glycosides, amino acids, sugars, vitamins, and other nutrients [3,4,5,6,7]. In recent years, due to a deeper understanding of its nutritional and bioactive components, the production of bitter gourd has gradually gained popularity worldwide. This has also sparked a growing interest in the study of the regulatory mechanisms governing the growth and development of bitter gourd within the field of horticultural crops.

Plant growth and development is an extremely complex process that encompasses the formation of various tissues and organs, color transition, and quality improvement. It entails physiological and biochemical reactions such as energy metabolism, hormone signal response, cell division, and enlargement. The entire process necessitates a multitude of genes and metabolites to fulfill their regulatory functions. In cucurbit crops, extensive research has been conducted on molecular regulatory factors related to fruit growth and development. For instance, in cucumber (*Cucumis sativus* L.), it was found that the fruit length and elongation are determined by locus of *fruit size 1.1* (*FS1.1)*, *FS3.2* and *FS3.3*, while fruit diameter development involves *FS1.2*, *FS2.1*, and *FS2.2* [8]. Further studies revealed that the RING-type E3 ligase short fruit 1 gene can modulate fruit elongation by regulating ethylene dosage in fruits through the ubiquitination-mediated degradation of 1-aminocyclopropane-1-carboxylate synthase 2 and itself [9,10]. *FRUITFULL* (*FUL*) allele *CsFUL1^A^* can also influence auxin accumulation in fruits by impacting the expression of *SUPERMAN*, *PIN-FORMED1* (*PIN1*) and *PIN7* genes, thereby affect fruit length [11]. Similarly, *CsSUN* plays a crucial role in shaping cucumber fruits by significantly affecting the expression of genes related to cell division, expansion, and auxin signal transduction [12]. For flesh thickness, a relevant locus, *fruit flesh thickness 2.1*, was finely mapped, and through sequence and expression analysis, *Csa2G058670* was inferred as the potential candidate gene [13]. In addition, *green flesh* (*gf*) loci *qgf5.1* and *qgf3.1* were identified as possibly associated with controlling flesh greenness, with *Csa5G021320* being the best candidate gene for *qgf5.1* [14]. In melon (*Cucumis melo* L.), a total of 40 QTLs related to fruit shape have been identified. Among these, it has been found that fruit diameter (FD) is affected by *FDQJ2.2*, and fruit length is related to the PI124112 allele, which may function to suppress shape elongation along with the SUN and ovate family protein genes [15,16,17,18,19]. Moreover, it has been reported that fruit shape (FS) is associated with genomic regions *FSQM1*, *FSQM2*, *FSQM8*, and *FSQM11*, and fruit weight (FW) is linked to *FWQM8* and *FWQM11*, suggesting that they are potential loci for genes that determine fruit morphology [20]. Interestingly, a 7638G/A-SNP in the *SEPALLATA2* (*SEP2*) gene was also found to potentially regulate fruit length [21]. Furthermore, it has been reported that the *ORANGE* gene, which controls chromoplast differentiation and carotenoid accumulation, leads to changes in fruit color [22,23]. In addition, numerous transcription factors, such as members of the NAC, MADS-box, NOR, WRKY, GH3 and bZIP families, also play crucial roles in the growth and development of melon [21,24,25,26,27,28,29,30]. In wax gourd (*Benincasa hispida* (*Thunb*.) Cogn), it has been found that fruit shape formation is associated with *Bch02G016830* and *BhSAUR60* [31,32]. Peel color is regulated by both *BhAPRR2* and *BhiPRR6*, because the former’s expression level is significantly lower in white-skinned fruits compared to green-skinned ones, and the latter is associated with flavonoid accumulation [33,34]. The above findings indicate that the growth and development of fruits are regulated by multiple genes, and different genes play distinct roles in the formation and development of various traits. This provides insights for the identification and analysis of regulatory genes during the growth and development of bitter gourd fruits.

The transcriptome reflects the expression of protein-coding genes, while metabolites are the end products of gene transcription–translation and serve as the material basis of an organism’s phenotype. Simultaneously, metabolic products can influence or regulate gene transcription, protein expression, and activity [35,36,37,38]. Integrated transcriptomic and metabolomic analysis is an experimental approach that enables the comprehensive profiling of both genes and metabolites. The combined analysis of these two omics allows for the exploration of various biological questions in plants from both the “cause” and “effect” perspectives [39,40,41,42]. Previously, a combined transcriptomic and metabolomic analysis uncovered the mechanisms behind pigment accumulation and cucurbitacin biosynthesis in the change in color of bitter melon fruit peel [43]. However, there is currently a limited number of analyses of other regulatory factors and their impact on the growth and development of bitter melon fruit. Consequently, this study employs a combined transcriptomic and metabolomic approach to elucidate significant regulatory factors and their roles in the growth and development process of bitter melon fruit, aiming to provide new insights for enriching and refining the regulatory network of growth and development in cucurbit crops. By integrating these two omics layers, we hope to identify key genes and metabolites that are crucial for controlling fruit size, shape, and quality—vital traits for enhancing the agricultural productivity and marketability of bitter melon and potentially other cucurbit species. This multidimensional analysis will offer a comprehensive understanding of the intricate interactions between genetic and metabolic pathways that govern plant growth and development.

## 2. Results

### 2.1. Identification of the Trends in the Growth and Development Patterns of Fruit

**Figure 1:**
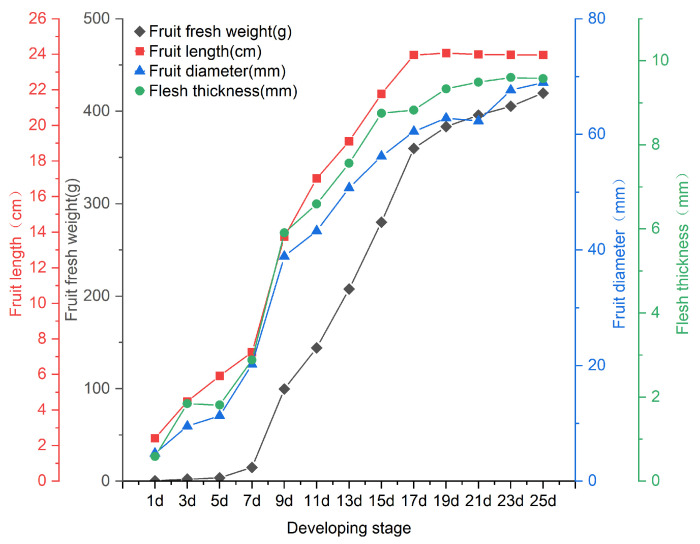
The variation patterns of fresh weight, fruit length, fruit diameter, and flesh thickness of bitter gourd fruit.

After measuring the fruit’s fresh weight, length, diameter, and flesh thickness every other day following pollination (Figure 1), we observed that the increase in these four traits was relatively slow during the first seven days, and this was followed by a rapid increase after the seventh day. A turning point was observed at 17 days, where the growth rate slowed once more, gradually stabilizing. The overall growth and development process follows a “slow–fast–slow” pattern. Consequently, 3 days (3 d), 10 days (10 d), 17 days (17 d), and 23 days (23 d) post-pollination were chosen for subsequent experiments. Then, it was found that all four traits observed in 2021 and 2023 also followed a similar growth pattern in these four stages (Table S1).

### 2.2. Overview of Transcriptome Analysis of Bitter Gourd Fruits in Different Growth and Development Stages

**Figure 2:**
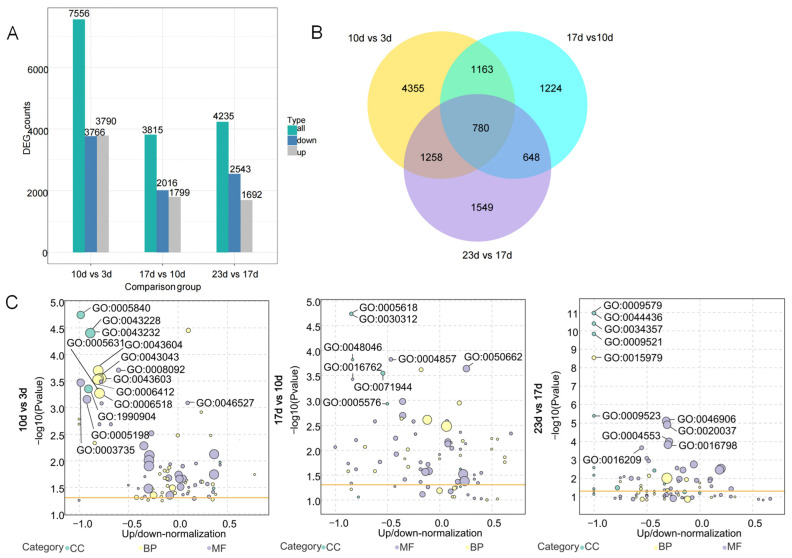
Transcriptome sequencing analysis results of bitter gourd fruit in different stages. (**A**) Up-/down-regulated and total DEG counts. (**B**) Venn diagram of DEGs. (**C**) GO enrichment analysis and ranking of the top five annotations. CC means cellular component, BP means biological process, and MF means molecular function.

Transcriptome sequencing yielded 18.91, 18.06, 18.1 and 18.09 Gb of clean bases at 3, 10, 17 and 23 d after fertilization, respectively. Each stage contained 46.58, 45.42, 46.75, and 47.78% of base G and C. In each stage, the Q30 values reached 92.61, 92.24, 92.26, and 92.64%, respectively. Of the total clean reads after quality control, 83.66 to 87.07% were unique matches with the reference genome (Table S2). In total, 3766 down- and 3790 up-regulated (7556) differentially expressed genes (DEGs) were found in the 10 vs. 3 d group, 2016 down- and 1799 up-regulated DEGs were found in the group 17 vs. 10 d group, and 2543 down- and 1692 up-regulated DEGs were found in the 23 vs. 17 d group (Figure 2A). Of the total DEGs, 4355, 1224 and 1549 were only identified in each group, while 780 showed up in all three groups (Figure 2B). Using a Padj < 0.05 threshold, 21, 8, and 14 important terms of Gene Ontology (GO) annotation were sequentially identified among the three groups. In the 10 vs. 3 d group, 973, 561, and 479 DEGs were found in the biological process, cellular component, and molecular function categories, respectively. Only 2 annotations, concerning the cellular carbohydrate metabolic process and glucosyltransferase activity, had more up- than down-regulated DEGs, while the other 19 annotations showed the opposite trend. From the top five DEGs ranked by quantity in each category, there were 13 annotations, with a ratio of the difference in number between up- and down-regulated DEGs to the total number of DEGs being −0.6 or less. These annotations are primarily associated with amide and peptide biosynthetic and metabolic processes, translation, non-membrane-bound organelles, ribonucleoprotein complexes, ribosomes and structural constituents, and structural molecule activity. In the 17 vs. 10 d group, 115 and 112 DEGs were identified in the categories of cellular component and molecular function, respectively, whereas no DEGs were found in the biological process category. Within the cellular component category, the majority of genes were down-regulated and associated with the cell wall, cell periphery, external encapsulating structure, extracellular region and apoplast. Annotations in the molecular functions category were primarily classified as coenzyme binding, enzyme inhibitor activity, and xyloglucan/xyloglucosyl transferase activity. Furthermore, coenzyme binding had the highest number of DEGs, and it was the only term with more up-regulated than down-regulated DEGs. In the 23 vs. 17 d group, 29, 131, and 299 DEGs were found in three categories (Table S2). Within the biological process category, no up-regulated DEGs were observed and all DEGs were associated with photosynthesis. For the cellular component category, DEGs were entirely down-regulated and primarily related to the thylakoid, photosynthetic membrane, photosystem, etc. In the molecular function category, the number of DEGs showed more up- than down-regulation, with the top five terms mostly related to tetrapyrrole binding, heme binding, hydrolase activity, antioxidant activity, etc. (Figure 2C, Table S3).

### 2.3. Overview of Metabolomic Analysis of Bitter Melon Fruits in Different Growth and Development Stages

**Figure 3:**
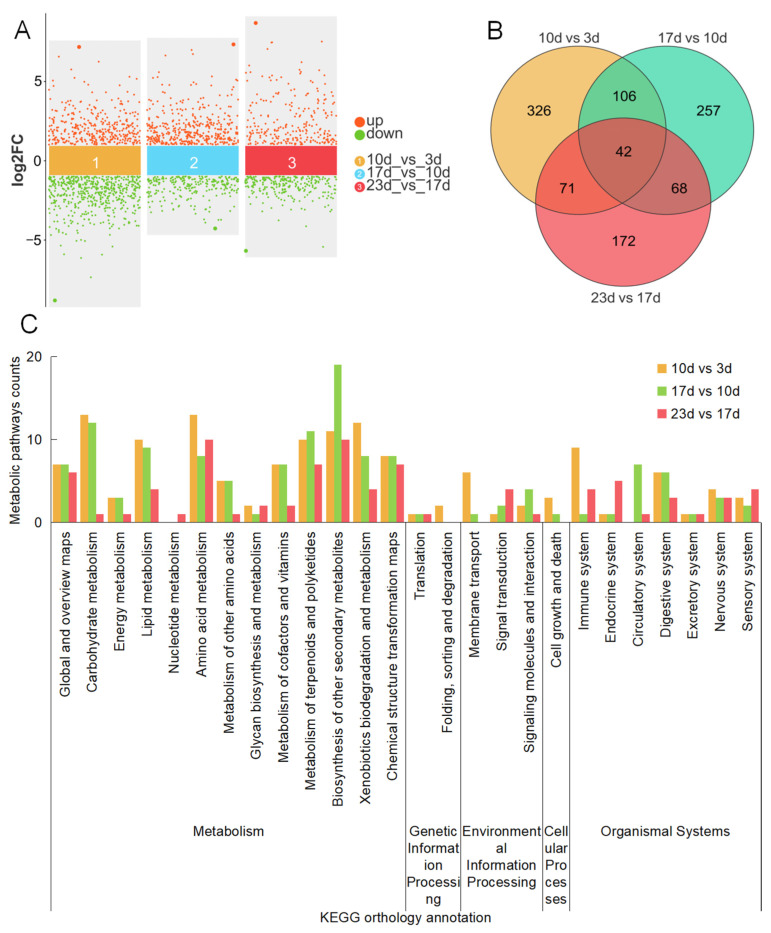
Metabolome analysis of bitter gourd fruit in different stages. (**A**) Up/down-regulated and total DEMs of 10 vs. 3 d, 17 vs. 10 d, and 23 vs. 17 d groups. (**B**) Venn diagram of DEMs. (**C**) Statistical analysis of KO annotation terms and categories.

In total, 1947, 1942, and 1927 differentially expressed metabolites (DEMs) were identified in the 10 vs. 3 d, 17 vs. 10 d, and 23 vs. 17 d groups, respectively. Among these, 896, 666, and 499 DEMs were found using a *p* value < 0.05 and a log2FoldChange value >1 as the threshold (Figure 3A). Furthermore, 545, 473, and 353 DEMs were sequentially identified in each group using a variable importance in the projection (VIP) value ≥ 1, with 326, 257, and 172 being unique to each group, while 42 DEMs were found in all three groups (Figure 3B). In these three groups, 385, 341, and 193 DEMs were in turn enriched in 141, 129, and 84 terms of KEGG Orthology (KO), which were involved in 26 categories across five classes including metabolism, genetic information processing, environmental information processing, cellular processes, and organismal systems (Table S4, Figure 3C). Among them, the class of metabolism had the most KO terms and DEMs in each group. Furthermore, in the 10 vs. 3 d group, the carbohydrate metabolism and amino acid metabolism categories both had the most KO terms, relating to 21 DEGs, with 13 up- and 8 down-regulated genes in the former, and 26 DEGs, with 10 up- and 16 down-regulated genes in the latter. In the 17 vs. 10 d group, the categories of biosynthesis of other secondary metabolites and carbohydrate metabolism were the top two in KO term counts, relating to 28 DEGs, with 24 up- and 4 down-regulated genes in the former, and 20 terms, with all being up-regulated genes in the latter. In the 23 vs. 17 d group, the categories of amino acid metabolism and biosynthesis of other secondary metabolites both had 10 KO terms, ranking first on the quantity leaderboard, relating to 12 DEGs, with 8 up- and 4 down-regulated genes in the former, and 23 terms, with 20 up- and 3 down-regulated genes in the latter. Furthermore, the categories of carbohydrate metabolism, amino acid metabolism, and biosynthesis of other secondary metabolites each play significant roles across various groups. KO terms within each category exhibit similarities. For instance, within carbohydrate metabolism, 13 and 12 terms were identified in the 10 vs. 3 d and 17 vs. 10 d groups, with 11 terms overlapping between both groups. In amino acid metabolism, a total of 10 terms were found in the 23 vs. 17 d group, which also appeared in the 10 vs. 3 d group. Within the biosynthesis of other secondary metabolites, 19 and 10 terms were identified in the 17 vs. 10 d and 23 vs. 17 d groups, with 8 terms being common to both groups. Additionally, some metabolites play roles in numerous metabolic processes, while others exhibit differences in specific pathways. For example, C00074 is involved in 22 metabolic pathways, whereas C09099 only participates in the flavonoid biosynthesis process. This indicates that the process of fruit growth and development is dynamic, with certain basic metabolites providing the material and energy foundation for the entire process. However, there are also substances with specificity that only play a major role in a particular stage, thus requiring comprehensive analysis.

### 2.4. Joint Transcriptome and Metabolome Analysis

**Figure 4:**
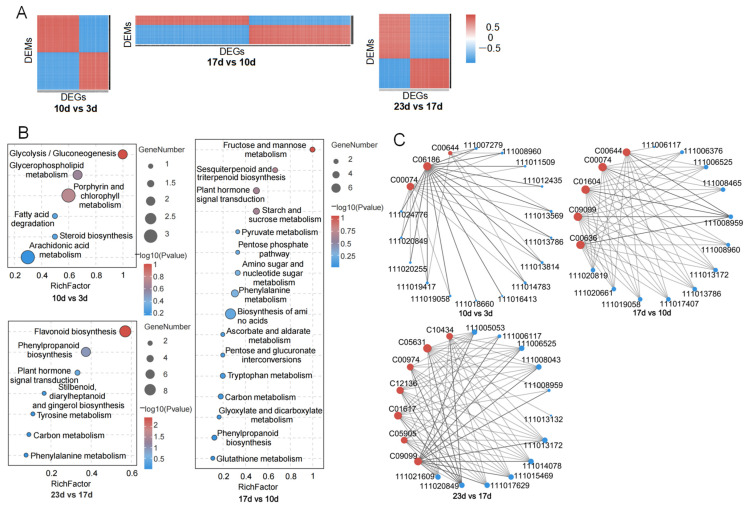
Joint transcriptome and metabolome analysis. (**A**) A correlation analysis of DEGs and DEMs of the 10 vs. 3 d, 17 vs. 10 d, and 23 vs. 17 d groups. (**B**) The co-enriched KEGG pathways in these three groups. (**C**) Network of important DEGs and DEMs identified from the glycolysis/gluconeogenesis pathway, fructose and mannose metabolism pathway, and flavonoid biosynthesis pathway, with a threshold of *p* < 0.05. The red dots represent DEMs, with the letters and numbers next to them indicating their KEGG IDs. The blue dots represent DEGs, and the numbers next to them are their gene IDs.

Correlation analysis was individually conducted on 7556 DEGs and 545 DEMs in the 10 vs. 3 d group, 3815 DEGs and 473 DEMs in the 17 vs. 10 d group, and 4235 DEGs and 353 DEMs in the 23 vs. 17 d group. The analysis revealed that 4162, 8832 and 12,125 types of relationship were found in each group with the condition of *p* value < 0.05 (Figure 4A, Table S5). In each group, 6, 16, and 7 co-enriched KEGG pathways were successively identified. Using a gene pathway *p* value of less than 0.05 as the threshold, three important pathways including glycolysis/gluconeogenesis, fructose and mannose metabolism, and flavonoid biosynthesis were found to have the minimal metabolite pathway *p* value but the maximal rich factor (Table S6, Figure 4B). Within these three pathways, a total of 76 important DEGs and 12 important DEMs were identified (Table S7). Furthermore, in the 10 vs. 3 d group, 34 types of correlations consisting of 15 positive and 19 negative correlations among 15 DEGs and 3 DEMs were detected at a *p* value < 0.05. C06186 was positively correlated with all 15 DEGs, C00074 was negatively correlated with 13 DEGs, and C00644 was negatively correlated with only 6 DEGs. Conversely, six DEGs were relevant to all three DEMs, seven DEGs were relevant to two DEMs, while two DEGs were relevant to only one DEMs. In the 17 vs. 10 d group, 54 types of correlations consisting of 5 positive and 49 negative correlations were similarly found between 12 DEGs and 5 DEMs. C00074, C00636, and C09099 were each related to 11 DEGs, and C00644 and C01604 were each related to 10 DEGs. By contrast, nine DEGs had one positive correlation and eight negative correlations with all five DEMs, and two DEGs and one DEG were negatively correlated with four DEMs and one DEM, respectively. In the 23 vs. 17 d group, 71 types of correlations consisting of 16 positive and 55 negative correlations were also discovered between 12 DEGs and 7 DEMs (Table S7, Figure 2C). Specifically, C05631 was related to 12 DEGs, C01617 and C09099 were related to the same 11 DEGs but with opposite positive and negative properties, C12136 was related to 10 DEGs, and C00974 and C10434 were each related to 9 DEGs. On the other hand, 9 DEGs were related to all 7 DEMs, and 8 DEGs showed the same positive and negative correlation properties while 1 DEG (ID:111020849) showed the opposite; each had 1 DEG associated with 4, 3, and 1 DEMs, respectively. This indicates that the relationship between genes and metabolites is not singular; one gene can be involved in the changes in multiple metabolites, and one metabolite may also be influenced by multiple genes.

### 2.5. The Expression of Important DEGs and DEMs in the Co-Enriched Pathway

**Figure 5:**
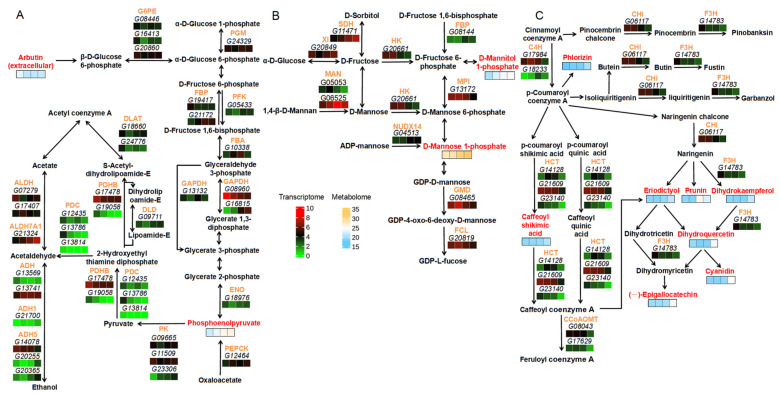
Expression differences in significant DEGs and DEMs in co-enriched pathways: (**A**) glycolysis/gluconeogenesis pathway, (**B**) fructose and mannose metabolism pathway, and (**C**) flavonoid biosynthesis pathway. Bold font indicates DEGs, enzymes, and DEMs involved in metabolic pathways; italic font represents DEMs; orange font indicates enzymes encoded by genes; and red font signifies important DEMs. Bars in shades of green to red comprise the heatmap of DEG expression, while bars in shades from blue to orange represent the heatmap of DEM expression.

In total, 52 DEGs and 12 DEMs were identified after comprehensive consideration of expression differences and importance, with an absolute log2FoldChange value of greater than 1, and another metabolite nearby was also employed to explain the expression network of the three important co-enriched pathways above. In the glycolysis/gluconeogenesis pathway, two DEMs—phosphoenolpyruvate (PEP) and arbutin (ARB)—exhibited opposite expression trends. This indicates that the former increases while the latter gradually decreases as development progresses. A total of 33 DEGs were found to display different expression trends from stages 3 to 10 d. Among these—24 genes, including *G05433*, encoding 6-phosphofructokinase 1 (PFK), G13569, encoding alcohol dehydrogenase (ADH), *G07279*, encoding aldehyde dehydrogenase (ALDH), *G09711*, encoding dihydrolipoyl dehydrogenase (DLD), G18976, encoding enolase 1/2/3 (ENO), *G19417*, encoding fructose-1,6-bisphosphatase I (FBP), *G24329*, encoding phosphoglucomutase (PGM), *G08960* and *G13132*, both encoding glyceraldehyde 3-phosphate dehydrogenase (GAPDH), *G17478* and *G19058*, both encoding pyruvate dehydrogenase E1 component subunit beta (PDHB), *G18660* and *G24776*, both encoding pyruvate dehydrogenase E2 component/dihydrolipoyllysine-residue acetyltransferase (DLAT), *G09665* and *G11509*, both encoding pyruvate kinase (PK), *G08446*, *G20860*, and *G16413*, all encoding glucose-6-phosphate 1-epimerase (G6PE), *G12435*, *G13786* and *G13814*, all encoding pyruvate decarboxylase (PDC), and *G14078*, *G20255* and *G20365*, all encoding S-(hydroxymethyl)glutathione dehydrogenase/alcohol dehydrogenase (ADH5), were found to be reduced. By contrast, nine genes, including *G13741*, encoding ADH, *G17407*, encoding ALDH, *G21172*, encoding FBP, *G16815*, encoding GAPDH, *G23306*, encoding PK, *G21700*, encoding alcohol dehydrogenase class-P (ADH1), *G21324*, encoding aldehyde dehydrogenase family 7 member A1 (ALDH7A1), *G10338*, encoding fructose-bisphosphate aldolase class I (FBA), and *G12464*, encoding phosphoenolpyruvate carboxykinase (PEPCK), were found to be increased. In the fructose and mannose metabolism pathway, two DEMs—D-Mannose 1-phosphate (ME-1P) and D-Mannitol 1-phosphate (MT-1P)—consistently maintained an upward trend. Among the 10 genes involved, i.e., *G08144*, encoding FBP, *G04513*, encoding ADP-sugar diphosphatase (NUDX14), *G11471*, encoding L-iditol 2-dehydrogenase (SDH), *G05053*, encoding mannan endo-1,4-beta-mannosidase (MAN), and *G20849*, encoding xylose isomerase (XI), showed up-regulated expression from stages 10 to 17 d. By contrast, *G06525*, encoding MAN, *G20819*, encoding GDP-L-fucose synthase (FCL), *G08465*, encoding GDP-mannose 4,6-dehydratase (GMD), *G20661*, encoding hexokinase (HK), and *G13172*, encoding mannose-6-phosphate isomerase (MPI), showed down-regulated expression. In the flavonoid biosynthesis pathway, a total of eight DEMs were identified. Among these, phlorizin (PHL), caffeoylshikimic acid (CSA), and (-)-Epigallocatechin (EGC) exhibited a continuously increasing expression. However, the other five DEMs displayed varying trends. Specifically, the expression of dihydrokaempferol (C00974), dihydroquercetin (TAX), eriodictyol (ERI), and cyanidin (CYA) increased from stages 17 to 23 d, while that of prunin (PRU) decreased. Nine related DEGs, including *G06117*, encoding chalcone isomerase (CHI), *G14783*, encoding naringenin 3-dioxygenase (F3H), *G08043* and *G17629*, both encoding caffeoyl-CoA O-methyltransferase (CCoAOMT), *G17984* and *G18233*, both encoding trans-cinnamate 4-monooxygenase (C4H), and *G14128*, *G21609* and *G23140*, encoding shikimate O-hydroxycinnamoyltransferase (HCT), were all down-regulated in expression during the period from 17 to 23 d. This indicates that as the growth and development of the fruit progress, the dominant metabolic pathways and the accumulated metabolites exhibit dynamic changes, and the expression of genes also varies to match the operational efficiency of the relevant enzymes during the whole process (Figure 5, Tables S8 and S9).

### 2.6. Relative Expression Trend of Important DEGs 

A total of 12 DEGs from three co-enriched pathways were randomly selected for an analysis of the relative expression trends using RT-qPCR (Table S10). Meanwhile, a comparison of these trends with the FPKM values obtained from RNA sequencing was performed (Figure S1). The analysis revealed that the trends derived from the two methods are essentially consistent. Among the seven DEGs from the glycolysis/gluconeogenesis pathway, six DEGs including *G05433*, *G07279*, *G16413*, *G13786*, *G19058* and *G20255* were down-regulated from stage 3 d to 10 d. Although *G07279* and *G20255* increased after 10 d, *G13786* and *G19058* continued to fall, and *G05433* and *G16413* exhibited a trend of first rising and then falling. *G10338* was up-regulated from stage 3 d to 10 d, then continued to rise before falling again. Among the three DEGs from the fructose and mannose metabolism pathway, *G11471* was up-regulated from 3 d to 23 d while *G05053* decreased at 23 d following an increase from 3 d to 17 d. *G06525* was down-regulated from stage 10 d to 17 d, but showed an upward trend before 10 d, followed by a continuous decline. In the flavonoid biosynthesis pathway, the expression of *G14783* roughly showed a sharp decrease from 3 d to 10 d and then remained steady for the rest of the time course experiment. *G18233* increased from 3 d to 17 d and decreased from 17 d to 23 d. The above findings not only demonstrate the accuracy of the transcriptome sequencing results, but also indicate that the regulation of genes on fruit growth and development is not static.

### 2.7. Correlation Analysis and Heat Network of DEGs, DEMs and Fruit Traits

**Figure 6:**
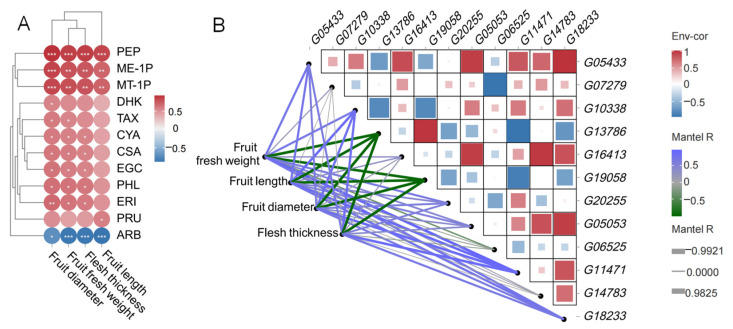
The correlation and heat network among DEGs, DEMs and fruit traits. (**A**) The correlation between DEMs and fruit traits. (**B**) The heat network of genes and fruit traits. The heatmap depicts the correlation between the relative expression levels of DEGs. Each square within the grid represents the sign of the correlation coefficient between DEGs, with the size of the color block indicating the magnitude of the absolute value of the correlation coefficient. The network graph represents the correlation between gene expression levels and various traits. The thickness of the lines indicates the strength of the correlation, and the color of the lines indicates the sign of the correlation coefficient. “*” indicates a significance level of *p* < 0.05, “**” indicates a significance level of *p* < 0.01, and “***” indicates a significance level of *p* < 0.001.

The correlation analysis between fruit traits and DEMs shows that, as a comprehensive trait, fruit fresh weight was relevant to the most DEMs, meaning that it is not only significantly negatively correlated with ARB, but also significantly positively correlated with all 10 DEMs except PRU. Fruit length involves the least DEMs, meaning that it is significantly negatively correlated with ARB and significantly positively correlated with PEP, ME-1P, MT-1P, and PRU. In other words, PEP, ME-1P, MT-1P and four growth traits are all significantly positively correlated, while ARB is significantly negatively correlated. PRU is only significantly positively correlated with fruit length. Correlation analysis between genes, as well as between genes and fruit traits, was conducted at the same time. On one hand, several genes exhibited a strong correlation with others, with *p* values less than 0.05 and correlation coefficients greater than 0.6 or less than -0.6. For instance, among the seven genes from the glycolysis/gluconeogenesis pathway, *G05433*, *G07279*, *G10338*, *G16413*, *G13786*, *G19058* and *G20255* were significantly related to each of the other 8, 1, 6, 6, 4, 6, and 4 genes, respectively. Among them, *G13786* was extremely significantly and strongly positively correlated with *G19058*, and they were both significantly negatively correlated with the same three genes: *G05433*, *G10338* and *G20255*. *G16413* was only correlated with *G05433*, but in addition, *G05433* also showed a significant positive correlation with *G10338*. Among the three genes from the fructose and mannose metabolism pathway, *G05053* was significantly positively related to six genes, *G06525* was significantly negatively related to two genes, and *G11471* was extremely significantly negatively related to two genes and significantly positively related to five genes. Among these, *G05053* was significantly positively related to *G11471*, whereas *G6525* did not show a significant relationship with either of them. *G14783* and *G18233*, both from the flavonoid biosynthesis pathway, exhibited strong positive correlations with four other genes of the former, and strong correlations with the eight other genes, including six positive correlations and two negative correlations of the latter. Among these, the correlation between *G14783* and *G18233* was extremely significant and positive. On the other hand, eight genes showed a significant correlation with fruit fresh weight, fruit length, fruit diameter, and flesh thickness, with *p* values less than 0.05. Genes *G05433*, *G10338*, *G11471*, and *G18233* were positively correlated with all four fruit traits, whereas G13786 and G19058 were negatively correlated with all traits. *G20255* was positively related to fruit fresh weight, fruit diameter, and flesh thickness, while *G05053* was only related to fruit length. The above results indicate that metabolites and genes have both positive and negative regulatory effects on the growth and development of fruit, and the relationships vary from distant to close, with regulatory effects ranging from strong to weak (Figure 6, Table S11).

## 3. Discussion

### 3.1. Growth and Development Pattern of Bitter Gourds

The growth and development stage of fruit begins with pollination and fertilization, after which the ovary starts to enlarge until the fruit is fully mature [44]. During this period, changes in traits such as fruit length, fruit diameter, flesh thickness, and fresh fruit weight not only affect the size and morphological development of the fruit but are also key factors in determining the final quality and yield. This study found that the changes in the fresh weight, length, and diameter of the fruit, as well as the flesh thickness of bitter melon, follow a similar “S”-shaped growth pattern with development, which can be roughly divided into three stages: The first is from the day of pollination to 7 days, during which the early development of the fruit is slow, and the changes in the four traits are relatively gentle. The second is from 7 to 17 days post-pollination, when the fruit enters the mid-term enlargement and development phase, with a rapid growth speed; the increase in the four traits is also very rapid. The third is 17 days post-pollination, during which the fruit’s growth rate slows once more and the fruit length remains nearly constant, while the other three traits exhibit minor increases and approach stabilization. This suggests that the fruit is gradually ripening and beginning to change color, indicating that the accumulation of nutrients and the drying of seed matter are imminent. This is similar to the trend of many other horticultural crops such as cucumbers, pumpkins, and tomatoes, where fruit size and weight increase with development in a “slow–fast–slow” pattern [45,46,47,48], indicating that the growth and development of fruit in cucurbitaceous horticultural crops may follow similar rules.

### 3.2. Analysis of Major Metabolic Pathways and Related Regulatory Genes in Bitter Gourd Fruits During Different Growth and Development Stages

The growth and development of fruit require carbohydrates as the material and energy basis, and also require the synergistic regulation of hormones, enzymes, and environmental factors. Early studies have indicated that glycolysis/gluconeogenesis plays significant roles through nearly the entire process of plant growth and development, including seed germination in rice (*Oryza sativa* L.), maize (*Zea mays* L.) and carrot (*Daucus carota* L.) [49,50,51]; photosynthesis and growth of maize seedlings [52] and sugar and acid metabolism of cucumber fruits [53]; changes in the color of strawberry (*Fragaria × ananassa* (Weston) Duchesne ex Rozier) flesh [54]; polysaccharide biosynthesis of *Polygonatum cyrtonema* Hua rhizomes [55]; and improvements in grain yield in rice and wheat (*Triticum aestivum* L.) [56,57,58]. In this experiment, glycolysis/gluconeogenesis was found to play a major role in the early growth and development of bitter gourd fruit. For instance, a large amount of extracellular arbutin does not cause fluctuations in β-D-glucose 6-phosphate, and the down-regulation of *G20860*, *G08446*, *G16413*, and *G24329* may affect the efficiency of G6PE and PGM, influencing the generation of α-D-glucose 6-phosphate. However, α-D-glucose 6-phosphate can fortunately be supplemented from extracellular D-glucose and α-D-glucose, ensuring that subsequent metabolism proceeds normally. Then, during the α-D-glucose 6-phosphate-to-glycerate 2-phosphate process, *G19174* is up-regulated and *G21172* is down-regulated, but both encode FBP, achieving an internal balance without affecting enzyme function. A similar situation also includes G08960 and *G16815*, both of which encode GAPDH. Additionally, this process also involves the pentose phosphate pathway and carbon fixation by the Calvin cycle. Even with changes in the expression of some genes, compensation can be obtained from these two pathways to ensure the normal progression of the process. The accumulation of PEP mainly depends on the up-regulation of G12464 expression, while the expression changes in G23306, G*11509*, and *G09665* reach an internal balance, maintaining the efficiency of PK, resulting in no significant change in pyruvate content. During the pyruvate-to-acetyl coenzyme A process, *G17478*, *G19058*, *G13786*, *G12435*, and *G13814* are down-regulated, but there is no change in other genes encoding enzymes in this pathway, and this process also participates in the citrate cycle, indicating that it can be supplemented, ensuring the formation of acetyl coenzyme A and, subsequently, the normal supply of acetate. The balanced coordination of genes encoding aldehyde dehydrogenase family members, *G17407*, *G07279* and *G21324*, ensures the normal synthesis of acetaldehyde, reflecting that its synthesis mainly comes from this pathway rather than the PDC pathway, as the three genes encoding PDC, *G13786*, *G12435* and *G13814*, are down-regulated. Next, genes related to different alcohol dehydrogenases, including ADH, ADH1, and ADH5, are up-regulated (*G13741*, *G21700*) and down-regulated (*G13569*, *G20255*, *G20365*, *G14078*) in a dynamic balance, ensuring the stability of the redox reaction between acetaldehyde and ethanol. Therefore, it is inferred that glycolysis and gluconeogenesis, as critical processes in carbohydrate metabolism and energy production, play a dominant role in regulating the early growth and development of bitter gourd fruit. A single metabolite change cannot affect the occurrence of metabolic processes, while the dynamic expression and regulation of related genes play an important role in maintaining the smooth progress of metabolic processes and further growth and development.

Carbohydrates play a crucial role in the expansion of fruit flesh cells and the subsequent development of weight and quality. During the ripening and softening of watermelon (*Citrullus lanatus (Thunb.*) Matsum. & Nakai) and banana (*Musa banana* Lour.), several DEGs were primarily enriched in the fructose and mannose metabolism pathway [59,60]. In rambutan (*Nephelium lappaceum* L.), the activation of the fructose and mannose metabolism pathways leads to the accumulation of three dominant sugars (sucrose, fructose, glucose) from the half- to full-ripe stages, which predominantly accounts for the variation in taste [61]. In plum (*Prunus salicina* Lindl.), after 21 and 28 days of melatonin treatment, the DEGs accelerated fructose and mannose metabolism during the second stage of rapid fruit expansion [62]. In the context of the fruit expansion and ripening stages of apples (*Malus domestica* L.), with applications of 300 and 600 kg·hm^−2^ of urea, the DEGs were enriched in fructose and mannose metabolism 150 days after bloom [63]. These studies indicate that fructose and mannose metabolism plays a significant role in the growth, development, and maturation of fruits, ultimately affecting yield and quality formation. In our study, fructose and mannose metabolism was also found to be the key pathway in bitter gourd fruit from 10 to 17 d after pollination, during which the fruit was growing mostly rapidly. Moreover, different genes and metabolites within the pathway also exhibit differential expression. For example, the accumulation of MT-1P likely occurs through mutual transformation with D-fructose 6-phosphate, which is derived from the substantial decomposition of D-fructose 1,6-bisphosphate under the high expression of *G08144*, serving as an alternative source from D-fructose, because this process might be impacted by the decrease in D-fructose 6-phosphate acquisition from D-mannose due to the down-regulation of *G20661*. Conversely, to prevent the large-scale retention of D-fructose, genes *G11471* and *G20849* were increasingly expressed to ensure the high efficiency of SDH and XI in the conversion of D-fructose with D-sorbitol and α-D-glucose, thereby ensuring the stable progression of subsequent metabolic processes. 1,4-β-mannan reacts with H_2_O to produce D-mannose and ATP, through the action of MAN. Two encoding genes, *G05053* and *G06525*, one down-regulated and the other up-regulated, achieve a complementary balance, thus ensuring the smooth progress of this process. Then, under the influence of the lower expression of *G20661*, the production of D-mannose 6-phosphate from D-mannose decreases, while the down-regulation of *G13172* reduces the interconversion efficiency between D-mannose 6-phosphate and D-fructose 6-phosphate, ensuring a normal reaction between D-mannose 6-phosphate and ME-1P. Therefore, the accumulation of ME-1P originates from the extensive hydrolysis of ADP-mannose, a process that occurs under the up-regulated expression of *G04513*, as well as from the reduced downstream metabolic efficiency caused by the down-regulated expression of *G08465* and *G20819*. Thus, this indicates that during the rapid growth process, the fruit communicates through the differential expression of coding genes to dynamically recruit enzymes with different functions to perform their tasks, thereby achieving different levels of efficiency, thus forming an optimal regulatory network and levels.

Research on flavonoid biosynthesis has currently been reported in various crops, revealing that flavonoids are an important class of secondary metabolites widely found in plants, contributing to plant growth and development [64]. It was found that flavonoids originate from phenylpropanoid metabolism, specifically from the formation of p-coumaroyl-CoA through the catalysis of C4H on cinnamoyl-CoA in the ‘core phenylpropanoid pathway’. They not only influence the production of various products such as lignin and suberin but also likely serve as a bridge connecting subsequent flavonoid metabolism, acting as a general precursor [65,66,67]. On one hand, a classic route demonstrates that p-coumaroyl-CoA is first transformed into p-coumaroyl shikimate through esterification by HCT, and then converted into CSA via hydroxylation by C3H. Subsequently, caffeoyl-CoA is produced by the de-esterification of HCT, and ultimately, with the aid of caffeic/5-hydroxyferulic acid O-methyltransferase (COMT I) and CCoAOMT, ferulic acid and feruloyl-CoA are generated. These are important precursors of suberin and various lignins [68,69]. Meanwhile, in citrus, CsCCoAOMT1 favors flavonoids over caffeoyl-CoA and esculetin, efficiently methylating 6-, 7-, 8-, and 3′-OH of a wide array of flavonoids with vicinal hydroxyl groups, with a strong preference for quercetin (flavonol) and flavones [69]. On the other hand, the entry of p-coumaroyl-CoA into the flavonoid biosynthesis pathway triggers the synthesis of various specific flavonoids [70,71,72], such as PHL, isoliquiritigenin and naringenin chalcone. The latter can be converted into naringenin, which serves as the precursor for ERI, PRUn, DHK, TAX, CYA, EGC, etc. Throughout the process, it was observed that the expression levels of CHI and C4H are associated with the content of flavonoids and lignin in *Arabidopsis thaliana* [73,74]. The overexpression of *DcCHI1* or *DcCHI4* leads to increased flavonoid accumulation in tobacco (*Nicotiana tabacum* L.) [75]. HCT genes are correlated with phenolic acid levels in potato (*Lycopersicon esculentum* Mill.) tubers, and the abundance of F3H transcripts generally correlates with flavonoids [76,77]. Two CCoAOMT genes (*Cs1g22450* and *Cs8g05410*) in citrus (*Citrus reticulata* Blanco.) showed markedly high expression in early development stages, which coincided with the rapid accumulation of polymethoxylated flavones during this period [69]. In this experiment, flavonoid biosynthesis played a significant role during the 17–23 d stage. Many flavonoids, including PHL, EGC, and CYA, showed substantial accumulation by 23 d, while the expression levels of HCT, CHI, and CCoAPMT genes (*G21609*, *G06117*, *G08043*, and *G17629*) were all down-regulated. PRU, identified as an inhibitor of growth [78], reached its peak accumulation at 17 days and was present in significantly higher amounts than in other periods, indicating that growth was about to cease at 17 days and preparations for ripening had begun. That is, the fruit was approaching a transition in color and texture, requiring substantial enzyme activation and catalysis to produce the metabolites needed for later stages. Consequently, the expression of related genes was relatively efficient at this time. By 23 days, the fruit gradually began to ripen, with most metabolites having completed their accumulation. Thus, the role of these enzymes gradually diminished, and gene expression also weakened. Ultimately, this phenotypically aligned with the observed slow fruit growth after 17 days and minimal changes in traits. Additionally, at 23 days, the accumulation of color metabolites such as PRU, responsible for the variation in internode color of Populus violascens Dode [72], can produce naringenin, which was found to be more abundant in citrus fruits [79,80]; EGC and CYA, which are involved in anthocyanin biosynthesis, whereas anthocyanins constitute red, orange, blue, and purple pigments [66], are also likely the main reason for the orange–yellow coloration of the fruit upon maturity. This indicates that flavonoid biosynthesis is involved in developmental regulation during fruit ripening and plays a significant role in organ color changes throughout plant growth and development.

### 3.3. Analysis of Relative Expression Levels of Key DEGs and Their Correlation with Fruit Traits

Integrating the phenotypic characteristic with omics analysis significantly accelerates the identification of metabolism-related functional genes in plants. RT-PCR analysis and correlation analysis revealed that *G05433*, *G10338*, *G11471* and *G18233* showed significant positive correlations with all four growth traits, and G20255 exhibited significant positive correlations with three traits except fruit length, while *G05053* was significantly positively correlated with fruit length. In contrast, *G19058* and *G13786* demonstrated significant negative correlations with all four growth traits. This indicates that different genes have distinct regulatory effects on the growth and development of bitter gourd fruits. In early studies on banana fruit, it was found that PFK activity increased from preclimacteric to climacteric peak stages of ripening, and then slightly declined with further ripening [81]. In this experiment, the overall expression level of *G05433* encoding PFK was lower at 3 and 10 d compared to the latter two periods, suggesting that it may share similarities with gene regulation in bananas. In sugarcane (*Saccharum officinarum* L.), *ScPFK6* showed predominant expression in all tissues, whereas *ScPFK7*, *ScPFK8*, and ScPFK9, which belong to the monocot-specific subgroup, exhibited variations among species [82], indicating that PFK genes present a complex expression profile. Early detection revealed that the loss of *ZmFBA8* function reduced the growth of maize seedlings [83]; in tomato, the overexpression of *SlFBA7* enhanced the net photosynthetic rate, seed size, and stem diameter [84], whereas reduced *SlFBA7* expression led to a decrease in these traits, as well as dry weight [85]. In this experiment, the expression of gene *G10338* encoding FBA showed a continuous increase in bitter gourd fruits at 3, 10, and 17 days after pollination, followed by a decline at 23 days. These findings suggest that FBA genes positively impact plant growth and development, with a more pronounced effect during the early fruit growth stage compared to the maturation phase. The expression level of *G20255* encoding ADH was very low at 3 and 10 d, but high at 23 d, which is relatively consistent with previous research findings. This means that ADH genes, such as *SlscADH1* in tomato and *CmADH1*, *CmADH2*, *CmADH10*, *CmADH8* and *CmADH12* in melon, exhibit much lower expression levels during the young fruit stage compared to the late developmental or ripening stages. Previous studies found that PDH affects tobacco yield by influencing carbon partitioning between soluble carbohydrates and starch [86]. In sugar beet (*Beta vulgaris* L.), *bvPDH*_*E1alpha* encoding PDH E1 component subunit alpha is highly expressed in tap roots and flower buds [87]. However, *G19058*, which encodes the PDH E1 component subunit beta, shows peak expression at 3d of development in bitter gourd fruit, followed by a gradual decline, indicating that different subunits play similar roles in regulating early growth in tissues. The PDCs identified in oranges, pears, apples, and strawberries exhibit a ripening-related expression pattern [88,89,90,91]. The gene *PDC1* in melon was expressed at a higher level in ripe fruit, while *PDC2* expression was low in fruit and highest in the anther, pistil, ovary, and root tissues [92]. In this experiment, the expression level of *G13786* encoding PDC peaked at 3 d and then continued to decline, indicating that its sequence might be more similar to that of *PDC2* of the melon species than to that of *PDC1*, because *PDC1* participates in the biosynthesis of aromatic volatiles of fruit and is highly expressed in the late maturation stage [91], showing different expression patterns across diverse environmental conditions and in different plant organs. Coefficients between the expression level of *G11471* and each of the four traits were all above 0.9, indicating its substantial impact on fruit growth, which was largely consistent with earlier reports. For instance, SDH was found to play a crucial role in the synthesis of sorbitol, fructose, sucrose, and glucose, thereby supporting fruit growth, and knocking out might inhibit seedling development [93,94,95]. Additionally, its expression is dynamically regulated by the levels of the aforementioned polyols and sugars during fruit development and ripening, ultimately affecting fruit yield and quality [96,97,98,99]. MAN is associated with cell wall degradation and is a prerequisite for fruit softening in tomato and kiwifruit (*Actinidia chinensis* Planch) [100,101]. In this experiment, the expression level of *G05053* encoding MAN dynamically changes with the development of bitter melon fruit, suggesting that its regulation in plant growth and developmental events is flexible and adaptable. Previous research indicated that C4H belongs to the CYP73 family of the P450 superfamily and is the first key cytochrome P450 monooxygenase (P450) enzyme in the phenylpropanoid pathway [102,103]. In soybean (*Glycine max* (*Linn*.) Merr.), *GmC4H* genes display tissue- and developmental stage-specific expression patterns, and the *GmC4H20* transcript accumulation level increased gradually in early stages of seed development, peaked at 50 after pollination, and then decreased gradually during the late development stages [103]. In this experiment, the expression pattern gene *G18233* from bitter gourd fruit was similar, showing a continuous increase at 3 and 10 d, peaking at 17 d, and subsequently declining. This indicates that the C4H gene is present throughout the growth and development process of the fruit and maintains high-efficiency expression until maturity to accumulate sufficient pigment precursors for yellow coloration at ripening. The aforementioned results suggest that the growth and development of bitter gourd fruits are regulated by different genes with varying effects, while the same gene may play different roles in different plants depending on species, organs, growth stages, treatment conditions, and other factors.

## 4. Materials and Methods

### 4.1. Plant Materials and Fruit Trait Observation

High-generation inbred line materials from bitter gourds, named ‘ZK54’, were cultivated in the greenhouses at the Fuzhou Base in Fujian Province, China (119°30′ east longitude, 26°08′ north latitude), during the springs of 2020, 2021, and 2023. In 2020, from the second day after pollination, 5 bitter gourds with similar growth statuses were collected to observe the fruit traits, and then the same observation was carried out every other day. After measuring the length and fresh weight of the fruits, the fruits were cut horizontally at 2/3 of the distance from the fruit stem, to survey the diameter of the fruit and the width of the pulp cavity, and then to calculate the thickness of the flesh as follows: (fruit diameter—pulp cavity width) × 0.5. In 2021 and 2023, 5 fruits were also collected at 3 days (3 d), 10 days (10 d), 17 days (17 d) and 23 days (23 d) after pollination to perform the same observation each year.

### 4.2. Transcriptome Analysis

Nine bitter gourds were taken from each period of 2020, and three biological replicates were used to carry out RNA-sequencing at Vonuo Biotechnology Co., Ltd., located in Fujian, China. RNA was firstly extracted by Trizol (Invitrogen, Carlsbad, CA, USA). Then, after the total RNA met the requirements of a mass concentration >50 ng/μL, an RNA integrity value >7.0, OD260/OD280 >1.8, and a total RNA value > 1 μg, it was used to construct a library by using Illumina’s NEBNext^®^ UltraTM RNA Library Prep Kit( NEB, MA, USA). Then, Illumina HiSeq2500 and HISAT2 v2.0.5 were used for transcriptome sequencing and alignment (Momordica charantia ASM199503v1, https://ftp.ncbi.nlm.nih.gov/genomes/all/GCF/001/995/035/GCF_001995035.1_ASM199503v1/, accessed on 28 November 2020). The expression level of genes was calculated using the Fragments Per Kilobase of exon per Million reads mapped (FPKM) value, and differential gene screening was based on the “biological duplication” type. DESeq2 software (version: 1.16.1) and a negative binomial distribution *p*-value calculation model were used, with “|log2 (Fold Change)| > 0, padj < 0.05” as the standard. BLAST2GO software (version: 4.1.9) was used for Gene Ontology (GO) annotation, and KOBAS software (version: 2.0) was used for Kyoto Encyclopedia of Genes and Gnomes (KEGG) annotation. ClusterProfiler software (version: 3.4.4) was also used to perform GO functional enrichment analysis and KEGG pathway enrichment analysis on the differential gene set.

### 4.3. Metabolome Analysis

Nine mixed samples of bitter gourds were taken from each period of 2020, stored in liquid nitrogen, and sent to extract and analyze their metabolites using ultra-high-performance liquid chromatography–tandem mass spectrometry (UHPLC-MS/MS) at Biozeron Biotechnology Co., Ltd., located in Shanghai, China. In brief, the tissues were firstly lyophilized and pulverized, and 200 mg frozen powder was weighed and extracted with 0.6 mL 2-ChloroL-phenylalanine (4 ppm, formulated with methanol). Secondly, 300 µL supernatant was collected and filtered through a 0.22 µmol membrane filter after ultrasonic and centrifugal treatment. Thirdly, the filtrate was transferred into the detection vial and used to perform LC-MS analysis. Fourthly, an amount of 20 µL of filtrate from each tested sample was mixed to create a quality control (QC) sample. Finally, the entire data collection process was monitored by the method of interspersing QC samples in the sequence, which means that in the running sequence, 6 QC sample-balancing instruments were used (monitoring the pressure changes before and after each injection and the shift in the retention time of the main peaks in the TIC graph), and then one QC sample was inserted after every 10 detection samples.

The ACQUITY UPLC HSS T3 column (100 mm × 2.1 mm, 1.7 μm, Waters, Milford, USA) was used for chromatographic separation. The column temperature was 40 °C and the flow rate was 0.35 mL/min. Mobile phase A consisted of water and 0.1% formic acid, while mobile phase B consisted of acetonitrile. The elution gradient was as follows: 0–1.0 min, 5% B; 1.0–9.0 min, 5~100%B; 9.0–12.0 min, 1000%B; 12.0–15.0 min 5% B. The sample volume for each sample was 5 μL. The primary ion scanning range (m/z) of mass spectrometry was 80–1200, with a resolution of 70,000, the secondary fragmentation ion resolution was 17,500, and the energy gradient was 20/50/100.

An Ultimate 3000 Ultra-High-Performance Liquid Chromatography System (Dionex, Sunnyvale, CA, USA) in a Series Q-Exactive Quadruple Rod Electrostatic Field Orbital Trap High-Resolution Mass Spectrometer (Thermo Fisher Scientific, Woolsey, GA, USA) was used to collect data in positive and negative modes, respectively. In positive-ion mode, the sheath gas flow rate was 40 arb, the auxiliary gas flow rate was 10 arb, the spray voltage was 3.5 kV, the ion transfer tube temperature was 320 °C, and the ion source temperature was 300 °C; in the negative-ion mode, the sheath gas flow rate was 38 arb, the auxiliary gas flow rate was 10 arb, the spray voltage was 3 kV, the ion transfer tube temperature was 320 °C, and the ion source temperature was 300 °C. The primary mass spectrometry scan of the sample used a scan range of m/z 80–1200 and resolution of 70,000 FWHM, whereas the secondary scan used a resolution of 17,500 FWHM and an energy gradient of 20, 50, and 1000 eV.

Data processing and metabolite identification were performed using MetaboAnalyst 5.0 (https://www.metaboanalyst.ca/, accessed on 5 July 2023). This project employed Par-Scaling-formatted data for principal component analysis (PCA). The VIP (variable importance in the projection) values (threshold ≥ 1) from the PLS-DA model, combined with the *p*-values (*p*-value ≤ 0.05) from independent sample t-tests, were used to identify differentially expressed metabolites. The KEGG database was utilized to annotate metabolic pathways of the differential metabolites.

### 4.4. Joint Analysis of the Transcriptome and Metabolome

With the aim of identifying highly systematic changes occurring during the period of fruit growth and development, integrative analysis was conducted to explore the correlation between metabolite accumulation and gene expression. After calculating the Pearson correlation coefficient, using R software (version: 4.2.2), the significance of correlations was determined by setting a threshold with an absolute value of correlation coefficient value greater than 0.99 and a *p*-value less than 0.01. DEGs and DEMs were screened and common KEGG pathways were annotated. For these genes and metabolites, expression abundance and generate heatmaps were quantified.

### 4.5. Gene Expression Analysis via RT-qPCR

Bitter gourds were taken from each period of 2023 and stored in liquid nitrogen. RNA extraction, reverse transcription, and fluorescence quantitative analysis of fruits in different stages were performed using kits supplied by Novozymes Biotechnology Co., Ltd. (Nanjing, China). Primers were designed based on the sequences obtained from transcriptome sequencing analysis (Table S10). The reaction system consisted of 10 μL of 2 × ChamQ Universal SYBR qPCR Master Mix, 2 μL of cDNA, 0.4 μL of up- and downstream primers, and 7.2 μL of ddH_2_O. The reaction procedure was as follows: pre-denaturation at 95 °C for 30 s; denaturation at 95 °C for 10 s; annealing at 60 °C for 30 s and 40 cycles; and collection of melting curves at 95 °C for 15 s, 60 °C for 1 min, and 95 °C for 15 s. The experiment was repeated three times, and the relative expression level of genes was calculated using the 2^−ΔΔCT^ method.

### 4.6. Data Statistics and Analysis

All data were analyzed and visualized using Original 2021, SPSS Statistics 21.0, WPS Office, and OmicsShare Tools (https://www.omicshare.com/tools/Home/Soft/getsoft, accessed on 23 May 2024).

## 5. Conclusions

The growth and development of bitter gourd fruit are complex and systematic processes. In this experiment, we observed that the fruit exhibited a pattern of initial slow growth, followed by rapid development, and finally a plateau phase. Several differentially expressed metabolites and genes were identified through transcriptome and metabolome sequencing. Joint analysis of both sequencing datasets revealed the glycolysis/gluconeogenesis pathway, fructose and mannose metabolism pathway, and the flavonoid biosynthesis pathway in different-developmental-stage comparison groups, indicating their core position in early, middle, and late stages of fruit growth and development. Among these, arbutin showed a significantly negative correlation with all four growth traits, further confirming its role as a growth inhibitory factor. The coordinated up- and down-regulation balance among multiple genes encoding the same enzyme ensures normal enzymatic function, thereby facilitating the smooth progression of relevant pathways. Gene expression levels exhibited dynamic changes during fruit growth and development, showing varying correlations with growth traits. This indicates that the fruit requires the recruitment of different genes for differential expression to maintain normal metabolic activities, ultimately ensuring the successful completion of the growth and development process. This study provides evidence for improving our understanding of bitter gourd growth regulation mechanisms and offers a theoretical foundation for the future precision breeding of bitter gourd.

## Data Availability

The raw data supporting the conclusions of this article will be made available by the authors on request.

## Supplementary Materials

The following supporting information can be downloaded at https://www.mdpi.com/article/10.3390/plants14142248/s1: Table S1: Differences in fresh weight, fruit length, fruit diameter, and flesh thickness of bitter gourd fruit in different stages; Table S2: Statistical analysis of transcriptome data; Table S3: Statistical analysis of GO enrichment and differential expression genes; Table S4: Statistical analysis of KO annotation and differential expression metabolites; Table S5: Correlation analysis of DEGs and DEMs; Table S6: Transcriptome and metabolome co-enriched pathway analysis and screening; Table S7: Correlation analysis of 76 important DEGs and 12 important DEMs identified from glycolysis/gluconeogenesis, fructose and mannose metabolism, and flavonoid biosynthesis; Table S8: Expression trend of 52 important DEGs identified from three co-enriched pathways; Table S9: Expression trend of 12 important DEMs identified from three co-enriched pathways; Table S10: Gene primers for RT-qPCR analysis; Table S11: Correlation analysis of DEG relative expression levels and their relationship with fruit growth traits. Figure S1: Relative expression trends of 12 significant DEGs selected from three co-enriched pathways. Bars represent the relative expression-level changes calculated from RT-qPCR, while the line graph indicates the FPKM value changes obtained from RNA sequencing. The vertical axis on the left represents FPKM values, and the vertical axis on the right represents relative expression levels.
