# Supplementary material for: Transcriptomic and Metabolomic Joint Analysis Revealing Different Metabolic Pathways and Genes Dynamically Regulating Bitter Gourd (Momordica charantia L.) Fruit Growth and Development in Different Stages
Source: Plants (Basel). 2025 Jul 21;14(14):2248. doi: 10.3390/plants14142248 (PMC12299391; doi:10.3390/plants14142248)
Supplement: Supplementary file 1 [file plants-14-02248-s001.zip › plants-3640262-Supplementary Figure.pdf]

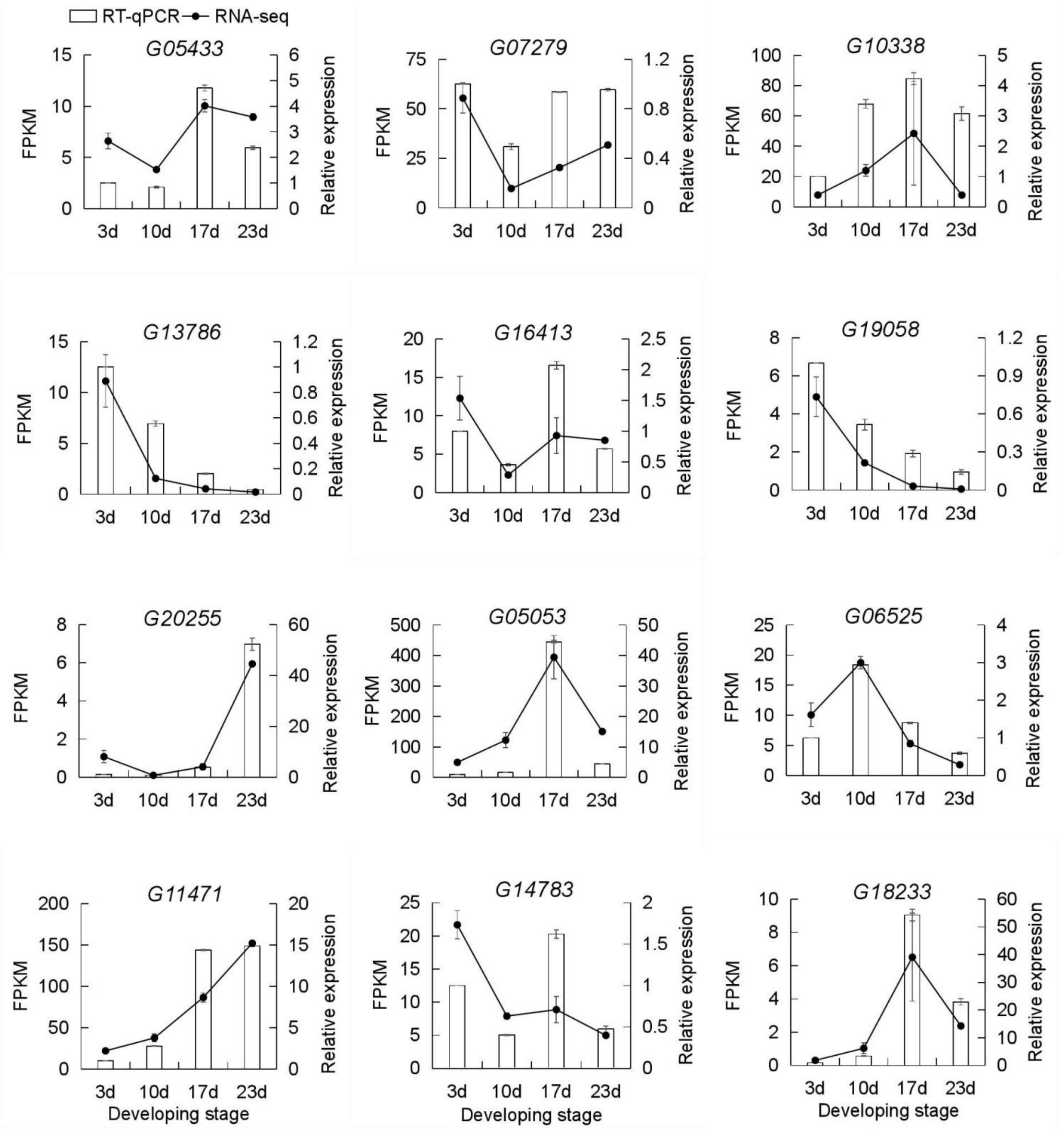

Figure S1. Relative expression trends of 12 significant DEGs selected from three co-enriched pathways. Bars represent the relative expression-level changes calculated from RT-qPCR, while the line graph indicates the FPKM value changes obtained from RNA sequencing. The vertical axis on the left represents FPKM values, and the vertical axis on the right represents relative expression levels.
